# A Risk Score for In‐Hospital Death in Patients Admitted With Ischemic or Hemorrhagic Stroke

**DOI:** 10.1161/JAHA.112.005207

**Published:** 2013-02-22

**Authors:** Eric E. Smith, Nandavar Shobha, David Dai, DaiWai M. Olson, Mathew J. Reeves, Jeffrey L. Saver, Adrian F. Hernandez, Eric D. Peterson, Gregg C. Fonarow, Lee H. Schwamm

**Affiliations:** 1 Department of Clinical Neurosciences, Hotchkiss Brain Institute, University of Calgary, Calgary, Alberta, Canada (E.E.S., N.S.); 2 Duke Clinical Research Institute, Durham, NC (D.D., D.W.M.O., A.F.H., E.D.P.); 3 Department of Epidemiology, Michigan State University, East Lansing, MI (M.J.R.); 4 Department of Neurology, University of California, Los Angeles, CA (J.L.S.); 5 Division of Cardiology, University of California, Los Angeles, CA (G.C.F.); 6 Stroke Service, Massachusetts General Hospital, Boston, MA (L.H.S.)

**Keywords:** intracerebral hemorrhage, ischemic stroke, mortality, subarachnoid hemorrhage

## Abstract

**Background:**

We aimed to derive and validate a single risk score for predicting death from ischemic stroke (IS), intracerebral hemorrhage (ICH), and subarachnoid hemorrhage (SAH).

**Methods and Results:**

Data from 333 865 stroke patients (IS, 82.4%; ICH, 11.2%; SAH, 2.6%; uncertain type, 3.8%) in the Get With The Guidelines—Stroke database were used. In‐hospital mortality varied greatly according to stroke type (IS, 5.5%; ICH, 27.2%; SAH, 25.1%; unknown type, 6.0%; *P*<0.001). The patients were randomly divided into derivation (60%) and validation (40%) samples. Logistic regression was used to determine the independent predictors of mortality and to assign point scores for a prediction model in the overall population and in the subset with the National Institutes of Health Stroke Scale (NIHSS) recorded (37.1%). The c statistic, a measure of how well the models discriminate the risk of death, was 0.78 in the overall validation sample and 0.86 in the model including NIHSS. The model with NIHSS performed nearly as well in each stroke type as in the overall model including all types (c statistics for IS alone, 0.85; for ICH alone, 0.83; for SAH alone, 0.83; uncertain type alone, 0.86). The calibration of the model was excellent, as demonstrated by plots of observed versus predicted mortality.

**Conclusions:**

A single prediction score for all stroke types can be used to predict risk of in‐hospital death following stroke admission. Incorporation of NIHSS information substantially improves this predictive accuracy.

## Introduction

Stroke is a leading cause of death in the United States, and a substantial portion of those deaths occur during hospitalization for acute stroke. The risk of death varies greatly across stroke types, with higher mortality from intracerebral hemorrhage (ICH) and subarachnoid hemorrhage (SAH) compared with ischemic stroke (IS).

Predicting risk of stroke death may be useful to objectively determine prognosis, target care to patients at risk, counsel patients and families regarding end‐of‐life decisions, and help clinicians and hospitals understand whether stroke case fatality rates are similar to expected rates based on patient case mix. Several risk scores have been published for prediction of mortality following IS^1–7^ and ICH,^8–16^ but there are few risk scores for SAH.^17–18^ There is only 1 validated risk score that has included both IS and ICH patients, to our knowledge.^1^ Limitations of many previously published models include derivation from small or selected populations and the use of noncontemporary cohorts, which may have limited applicability to current practice given the decreasing stroke case fatality rate of the past decade.

Get With The Guidelines—Stroke (GWTG‐Stroke) is a large national stroke registry and quality‐improvement initiative that has been previously used to derive and validate a risk score for prediction of death from IS.^19^ Participating hospitals now use this risk score, with Web‐based computerized decision support, to produce on‐demand reports of predicted and observed mortality in individual patients or groups of patients. However, there is a need to extend these predictions to patients with other stroke types. The objective of the current study was to derive and validate a more general predictive model for an individual patient's risk of in‐hospital death from acute stroke of any type (IS, ICH, SAH, or uncertain type).

## Methods

### Subject Population and Study Measurements

Characteristics of the GWTG‐Stroke program have been previously described.^20–21^ Participating hospitals used the Internet‐based Patient Management Tool (Outcome Sciences Inc, Cambridge, MA) to enter data, receive decision support, and obtain feedback on stroke quality of care. All hospitals entered consecutive patients with IS. Hospitals could enter ICH and SAH cases at their discretion; when they did so, they were instructed to submit consecutive cases, as for IS.^22^ Trained hospital personnel abstracted data using the Patient Management Tool with standardized data definitions and detailed coding instructions. The Internet‐based system performs checks to ensure that the reported data are complete and internally consistent. Hospital characteristics (ie, academic teaching status, bed size) were based on American Hospital Association data.^23^

Between October 1, 2001, and December 30, 2007, there were 1046 hospitals that contributed data on 408 412 stroke hospitalizations. We excluded patients transferred out to another acute care hospital (n=15 372, 3.8%) or transferred in from another acute care hospital (n=52 076, 12.8%). Furthermore, 7099 (1.7%) were excluded because of missing data on discharge destination, leaving 333 865 patients for analysis.

Each participating hospital received either human research approval to enroll cases without individual patient consent under the common rule or a waiver of authorization and exemption from subsequent review by their institutional review board. Outcome Sciences, Inc, serves as the data collection and coordination center for GWTG. The Duke Clinical Research Institute serves as the data analysis center and has institutional review board approval to analyze the aggregate deidentified data for research purposes.

### Statistical Analysis

First, the sample was randomly divided into a derivation sample (200 319, 60%) and a validation sample (133 546, 40%). Model building was carried out exclusively in the derivation sample. Using chi‐square tests for categorical variables and the Kruskal–Wallis test or Wilcoxon rank‐sum test for continuous variables, patient characteristics were compared among those who died in‐hospital or survived to discharge.

Variables for model inclusion were selected on the basis of prior literature, clinical relevance, and general availability and were selected in parallel with the model‐building process used to derive a risk score for predicting in‐hospital death from IS alone.^19^ These variables were entered into a multivariable logistic regression model to determine the independent predictors of in‐hospital death in the entire stroke population (including IS, ICH, SAH, and stroke of uncertain type). Age was entered as a continuous function if age >60 because we found a linear increased probability of death with each year of age >60 in prior analyses. The generalized estimating equations (GEE) approach was used to account for within‐hospital clustering.^24^ Stepwise backward elimination was used to remove nonsignificant variables (*P*>0.05) from the model.

Variables with missing data were imputed as follows: missing mode of arrival to the hospital (4.8%) was imputed to private transport (because ambulance personnel should have documented arrival times for patients arriving by ambulance), missing race (0.20%) was imputed to white, and missing arrival time (4.8%) was imputed to the off hours or weekend category (the most common category). The few patients with missing sex information (0.09%) were excluded from the models.

The beta coefficients from the final model were used to generate point scores for calculating mortality risk.^25^ The resulting mortality prediction rule was then validated by generating c statistics and plots of observed versus predicted mortality in the validation sample, using either 5 prespecified categories of predicted risk or 10 deciles of predicted risk. The c statistic was used as the primary measure of model discrimination.

Stroke severity, measured by the National Institutes of Health Stroke Scale (NIHSS), was not entered in the overall model because it was not routinely documented as part of routine clinical practice and was recorded for only 37.1% of patients. To test an a priori hypothesis that the NIHSS would be a strong determinant of mortality, we separately derived and validated a model in the subset of patients with the NIHSS documented, using the same approach described above.

Analyses were performed using SAS version 9.1.3 (SAS Institute, Cary, NC).

## Results

The study population consisted of 333 865 hospitalized stroke patients. Mean age was 71.1±14.7 years, and 53.3% were women. Admissions were submitted by 1046 hospitals. Median hospital bed size was 374 (interquartile range, 262 to 543), and 60.7% were identified as academic teaching hospitals according to American Hospital Association criteria.^23^

There were 274 988 (82.4%) with IS, 37 609 (11.2%) with ICH, 8664 (2.6%) with SAH, and 12 704 (3.8%) with stroke of uncertain type. The characteristics of patients according to stroke type are shown in Table 1. Overall, in‐hospital death occurred in 28 283 of 333 865 patients (8.5%). Mortality varied widely by stroke type: IS, 5.5%; ICH, 27.2%; SAH, 25.1%; uncertain type, 6.0% (*P*<0.001). Characteristics associated with in‐hospital death are shown in Table 2.

**Table 1.** Characteristics According to Stroke Type

Characteristic	Ischemic Stroke (n=274 988), %	Subarachnoid Hemorrhage (n=8664), %	Intracerebral Hemorrhage (n=37 509), %	Uncertain Type (n=12 704), %	*P* Value*
Age, y	74 (62, 83)	59.5 (49, 75)	73 (59, 82)	74 (61, 83)	<0.0001
Male	46.5	39.7	49.4	46.0	<0.0001
Race/ethnicity					<0.0001
White	73.9	67.8	68.4	70.6	
African American or Black	15.1	14.1	15.8	16.6	
Asian	2.3	4.0	4.4	1.8	
Hispanic	4.2	6.5	5.6	4.2	
Other	4.4	7.4	5.6	6.0	
Arrival mode to your hospital					<0.0001
EMS from scene	53.4	53.7	65.9	49.0	
Private transport	40.9	29.2	26.5	44.7	
Did not present via ED	5.7	17.1	7.6	6.3	
Initial NIHSS Score*	5 (2, 11)	3 (0, 15)	9 (3, 19)	3 (1, 8()	<0.0001
Medical history					
Atrial fibrillation	18.2	7.5	15.7	15.0	<0.0001
Atrial fibrillation, current admission	15.9	6.3	12.2	10.9	<0.0001
Prosthetic heart valve	1.5	0.9	1.6	1.3	<0.0001
Previous stroke/TIA	30.8	12.2	25.0	30.8	<0.0001
Coronary artery disease	27.5	13.6	21.1	25.0	<0.0001
Carotid stenosis	4.7	1.2	1.8	4.5	<0.0001
Diabetes mellitus	29.9	15.0	22.9	29.3	<0.0001
Peripheral vascular disease	5.2	1.8	3.4	4.7	<0.0001
Hypertension	74.0	54.8	71.4	69.8	<0.0001
Dyslipidemia	35.2	19.0	24.5	28.1	<0.0001
Smoker, current/past year	17.1	25.0	13.2	15.0	<0.0001
Arrived daytime regular hours*	46.8	33.1	40.8	45.5	<0.0001
Hospital characteristics					
Number of beds*	372 (262, 540)	434 (327, 587)	407 (281, 558)	317 (200, 499)	<0.0001
Teaching hospital*	60.6	69.0	63.4	50.0	<0.0001
Region					
Northeast	25.5	17.7	22.3	23.7	<0.0001
Midwest	19.9	17.5	17.4	16.9	
South	36.7	41.3	38.5	46.0	
West	17.9	23.6	21.8	13.4	
Outcome					
Died in the hospital	5.5	25.1	27.2	6.0	<0.0001

EMS indicates emergency medical services; ED, emergency department; NIHSS, National Institutes of Health Stroke Scale; TIA, transient ischemic attack. Age and NIHSS are reported as median (interquartile range).

* Significance testing by chi‐square test (for categorical variables) or Kruskal–Wallis test (for continuous variables).

* Available in 37.1% overall (ischemic stroke, 39.7%; intracerebral hemorrhage, 27.6%; subarachnoid hemorrhage, 15.8%; uncertain type, 23.5%).

* Daytime regular hours were defined as 7 am to 5 pm Monday to Friday; all other times (including all‐day Saturday and Sunday) were considered off‐hours.

* Missing in 0.90%.

* Missing in 0.99%.

**Table 2.** Characteristics of Stroke Patients Who Died in the Hospital

Characteristic	Overall (n=333 865)	Alive (n=305 582)	Dead (n=28 283)	*P* Value*
Age, y	74 (61, 82)	73 (61, 82)	78 (65, 85)	<0.0001
Male	46.7	46.9	44.3	<0.0001
Race/ethnicity				<0.0001
White	73.0	72.9	74.1	
African American or Black	15.2	15.5	12.6	
Asian	2.5	2.5	3.2	
Hispanic	4.4	4.4	4.1	
Other	4.9	4.8	6.1	
Stroke type				<0.0001
Ischemic stroke	82.4	85.0	53.5	
Subarachnoid hemorrhage	2.6	2.1	7.7	
Intracerebral hemorrhage	11.2	8.9	36.1	
Stroke of uncertain type	3.8	3.9	2.7	
Arrival mode to your hospital				<0.0001
EMS from scene	54.7	52.3	80.4	
Private transport	39.1	41.6	12.0	
Did not present via ED	6.2	6.1	7.6	
Initial NIHSS Score*	5 (2, 11)	4 (2, 10)	19 (12, 26)	<0.0001
Medical history				
Atrial fibrillation	17.5	16.6	26.8	<0.0001
Atrial fibrillation, current admission	15.1	14.4	22.4	<0.0001
Prosthetic heart valve	1.5	1.4	1.9	<0.0001
Previous stroke/TIA	29.6	29.8	27.7	<0.0001
CAD/prior MI	26.4	26.1	29.7	<0.0001
Carotid stenosis	4.3	4.4	3.0	<0.0001
Diabetes mellitus	28.7	29.0	26.2	<0.0001
PVD	4.9	4.9	5.5	<0.0001
Hypertension	73.0	73.2	71.0	<0.0001
Dyslipidemia	33.3	34.2	23.8	<0.0001
Smoker, current/past year	16.8	17.3	11.6	<0.0001
Arrived daytime regular hours*	45.7	46.3	40.2	<0.0001
Hospital characteristics				
Number of beds*	375 (262, 543)	373 (261, 543)	398 (267, 546)	<0.0001
Teaching hospital*	60.7	60.5	63.1	<0.0001
Region				
Northeast	24.9	24.8	25.7	<0.0001
Midwest	19.4	19.7	16.8	
South	37.4	37.5	35.7	
West	18.3	18.0	21.7	

EMS indicates emergency medical services; ED, emergency department; NIHSS, National Institutes of Health Stroke Scale; CAD, coronary artery disease; TIA, transient ischemic attack; MI, myocardial infarction; PVD, peripheral vascular disease.

* Significance testing by chi‐square test (for categorical variables) or Wilcoxon rank‐sum test (for continuous variables).

* Available in 37.1% overall (ischemic stroke, 39.7%; intracerebral hemorrhage, 27.6%; subarachnoid hemorrhage, 15.8%; uncertain type, 23.5%).

* Daytime regular hours were dened as 7 am to 5 pm Monday to Friday; all other times (including all‐day Saturday and Sunday) were considered off‐hours.

* Missing in 0.90%.

* Missing in 0.99%.

### Prediction Model Without Stroke Severity Information

**Figure 1.**
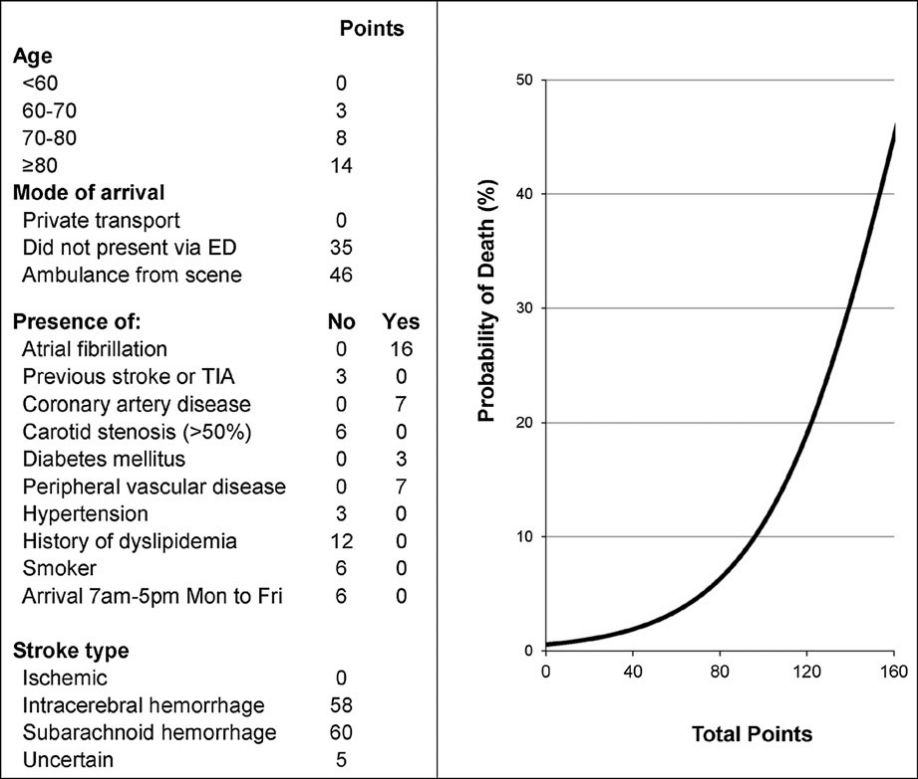
Prediction tool for in‐hospital death after admission for stroke. Risk of death is ≥45% for point score totals >160. 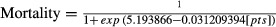. ED indicates emergency department; TIA, transient ischemic attack.

The derivation sample (n=200 319 patients, 60%) and validation sample (n=133 546 patients, 40%) were well matched with respect to patient characteristics and overall mortality, with the sole exception that history of hypertension was slightly more frequent in the derivation sample than in the validation sample (73.2% versus 72.9%, *P*=0.04). A logistic regression model for in‐hospital death was built using the following candidate predictor variables based on our previous work: age, stroke type, method of arrival at the hospital, history of atrial fibrillation, previous stroke, coronary artery disease, carotid stenosis, diabetes, peripheral vascular disease, hypertension, dyslipidemia, smoking, and weekend or night admission. All these candidate variables were significant independent predictors of mortality. The regression‐model beta coefficients were then used to derive point scores that could be used to predict a patient's risk of dying in the hospital (Figure 1). The predicted in‐hospital mortality according to point score category is tabulated in Table 3 and is plotted as a continuous function of the risk score in Figure 1.

**Table 3.** Predicted In‐Hospital Mortality According to Risk Score Category

Points	In‐Hospital Mortality (%)
0 to 20	<1.1
21 to 40	1.1 to 1.9
41 to 50	2.0 to 2.5
51 to 60	2.7 to 3.5
61 to 70	3.6 to 4.7
71 to 79	4.8 to 6.3
81 to 90	6.5 to 8.4
91 to 100	8.7 to 11.2
101 to 110	11.5 to 14.7
111 to 120	15.1 to 19.0
121 to 130	19.5 to 24.3
131 to 140	24.9 to 30.5
141 to 150	31.1 to 37.5
151 to 160	38.2 to 45.0
>160	>45.0

**Figure 2.**
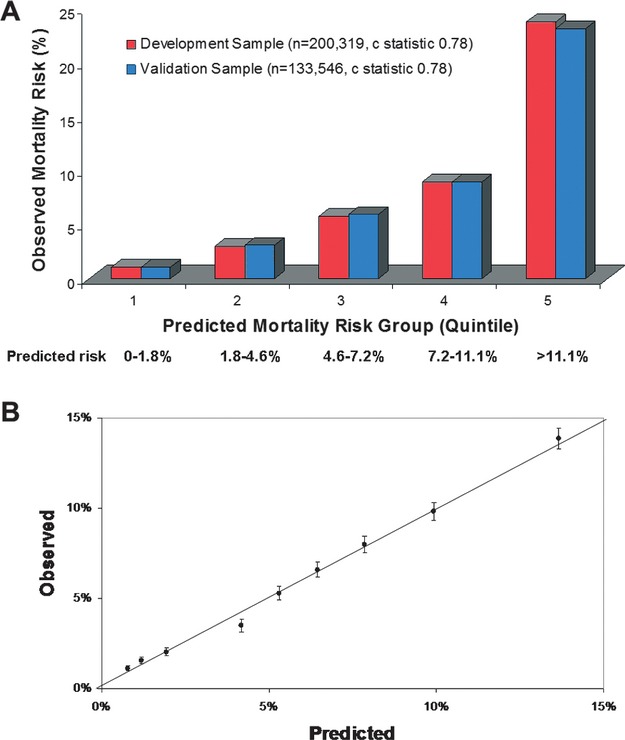
A, Observed vs predicted in‐hospital mortality according to quintiles of predicted risk. B, Observed (ie, actual) vs predicted in‐hospital mortality in the validation sample according to 10 deciles of predicted risk. The Hosmer–Lemeshow statistic was <0.001. Observed and expected mortality were highly correlated (*r*^2^=0.99).

The risk score demonstrated good discrimination in the reserved validation sample (c statistic, 0.78). Stratification of the derivation and validation samples by quintile of predicted in‐hospital mortality demonstrated the predicted risk of in‐hospital death varied >23‐fold across the quintiles, from 1.0% in quintile 1 to 23.2% in quintile 5 (Figure 2). A plot of observed versus predicted mortality in the validation sample, grouped into 10 deciles of predicted risk, showed excellent correlation between observed and predicted mortality (Figure 2). Despite this good correlation, the Hosmer–Lemeshow statistic was positive (*P*<0.001), indicating a statistically detectable difference between observed versus predicted events, possibly reflecting the high statistical power of the test because of the very large sample size.

### Prediction Model Including Stroke Severity Information (NIH Stroke Scale Score)

**Figure 3.**
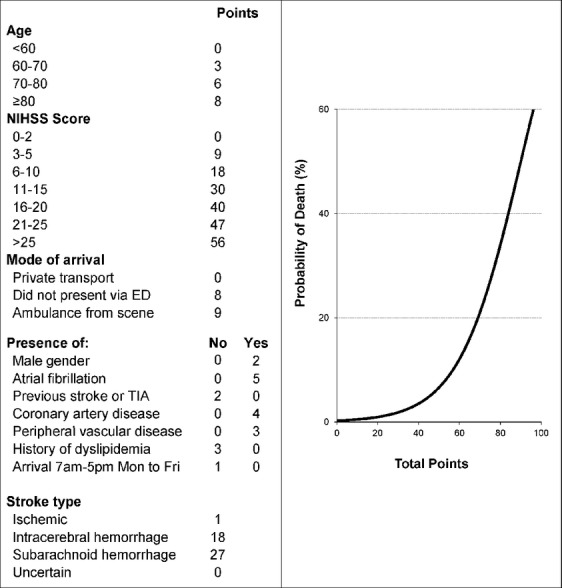
Prediction tool for in‐hospital death after admission for stroke, incorporating the NIH Stroke Scale score (NIHSS). The risk of death was ≥60.8% for point scores >100. 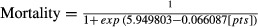.ED indicates emergency department; TIA, transient ischemic attack; NIH, National Institutes of Health.

**Figure 4.**
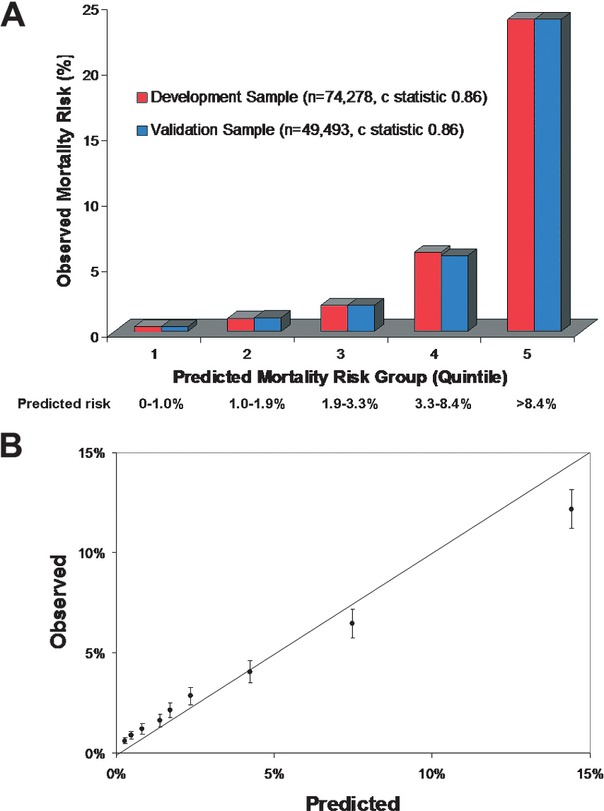
A, Observed vs predicted in‐hospital mortality, incorporating the NIH Stroke Scale score as a predictor, according to quintiles of predicted risk. B, Observed (ie, actual) vs predicted in‐hospital mortality in the validation sample according to 10 deciles of predicted risk. The Hosmer–Lemeshow statistic was <0.001. Observed and expected mortality were highly correlated (*r*^2^=0.99). NIH indicates National Institutes of Health.

We also derived a model in the subset of patients with NIHSS documented on admission (n=123 916, 37.1%). Documentation of NIHSS varied by stroke type: IS, 39.7%; ICH, 27.6%; SAH, 15.8%; uncertain type, 23.5% (*P*<0.001). As with our prior approach, 60% of the sample of patients with NIHSS recorded was used for derivation (n=74 278), and 40% was used for validation (n=49 483). The 2 samples were well matched with respect to patient characteristics and overall mortality, with no significant differences (data not shown). The NIHSS was strongly associated with mortality; median NIHSS was 19 in those who died (interquartile range, 12 to 26) compared with 4 in those who survived (interquartile range, 2 to 10; *P*<0.001). The c statistic for a model including the NIHSS as the only predictor was 0.84. Point scores for the full prediction model including the NIHSS are shown in Figure 3, and a table of predicted mortality according to point score category is shown in Table 4. The validation sample c statistic for the model including NIHSS (0.86) was greater than the c statistic for the model derived without NIHSS (0.78, *P*<0.001). Plots of observed versus predicted events showed good correlation (Figure 4), even though the Hosmer–Lemeshow test indicated a statistically significant difference between observed and predicted event rates (*P*<0.001).

**Table 4.** Predicted In‐Hospital Mortality According to Risk Score Category, Model Including NIH Stroke Scale Score

Points	In‐Hospital Mortality (%)
0 to 20	≤1.3
21 to 30	1.3 to 2.4
31 to 40	2.4 to 4.2
41 to 50	4.3 to 7.3
51 to 60	7.5 to 12.6
61 to 70	12.7 to 20.7
71 to 75	20.8 to 26.0
76 to 80	26.1 to 32.1
81 to 85	32.2 to 38.9
86 to 90	40.0 to 46.1
91 to 95	46.2 to 53.6
96 to 100	53.7 to 60.8
>100	>60.8

NIH indicates National Institutes of Health.

### Model Predictions in Each Stroke Type

Model discrimination is shown in Table 5, with and without incorporation of the NIHSS as a variable. Discrimination of in‐hospital death within each individual stroke type was worse than that in the overall model; this was not unexpected because stroke type was a powerful independent predictor of mortality in the overall model. When controlling for NIHSS, discrimination within each stroke type was excellent.

**Table 5.** Model Discrimination in Each Stroke Type

Stroke Type	Model			
	Without NIHSS		With NIHSS	
	c Statistic	H‐L Statistic	c Statistic	H‐L Statistic
All	0.78	<0.001	0.86	<0.001
Ischemic stroke alone	0.72	<0.001	0.84	<0.001
ICH alone	0.66	<0.001	0.82	<0.02
SAH alone	0.69	<0.001	0.89	<0.13
Uncertain	0.72	<0.02	0.88	0.007

NIHSS indicates National Institutes of Health Stroke Scale; H‐L statistic, Hosmer–Lemeshow statistic; ICH, intracerebral hemorrhage; SAH, subarachnoid hemorrhage.

## Discussion

Using data from a large nationwide study, we have generated a risk score that reliably predicts in‐hospital mortality in patients with IS, ICH, SAH, and stroke not classified. To our knowledge, this is the first validated clinical risk score for discrimination of death in all 3 major stroke types. When information on stroke severity was incorporated, as measured by the NIHSS, discrimination was substantially better. The NIHSS was the strongest determinant of in‐hospital death, more so than stroke type, even though stroke mortality is known to vary widely between IS, ICH, and SAH. Consequently, the risk score including NIHSS performed well within each individual stroke type. The risk score is complex but easy to use with the aid of Web‐based computational support and has already been implemented into the GWTG‐Stroke Patient Management Tool.

The calibration of the models appeared excellent, with high correlations between observed and expected event rates (Figures 2 and 4). However, the Hosmer–Lemeshow test was significant for most models, indicating a significant difference between the observed and predicted event rates. The most likely explanation for this discrepancy is that the statistical power of the Hosmer–Lemeshow test was excessively high because of the large sample sizes, with as many as 333 865 patients included in the models. The Hosmer–Lemeshow test is overly sensitive to trivial deviations from the ideal fit when the sample size is this large.^26^

The ability of this risk score to discriminate patients who died from patients who survived was similar to or better than previously published models. We used the c statistic as the primary measure of model discrimination. The c statistic is equivalent to the probability that the predicted risk of death is higher for patients who died than for patients who survived and is also equivalent to the area under the receiver operating characteristics (ROC) curve.^27^ The c statistic for our model without NIHSS information was 0.78 and for the model including NIHSS was 0.86. Previous prediction models for in‐hospital or 30‐day death following ischemic stroke have reported c statistics ranging from 0.79 to 0.88.^1–4,1–7,19^ Prediction models for in‐hospital or 30‐day death following ICH have reported c statistics ranging from 0.86 to 0.90.^8–16,28^ A single risk score for 90‐day mortality after SAH had an area under the ROC curve of 0.69 and 0.75 in 2 independent validation samples.^17^ A previous model of in‐hospital mortality in all forms of stroke (IS, ICH, SAH) based solely on administrative data had lower discrimination (c statistic, 0.72) than our model not including NIHSS (0.78) or including NIHSS (0.86).^29^ Our finding that the addition of NIHSS information resulted in improved model discrimination is consistent with a prior analysis of Get With The Guidelines‐Stroke data, which showed that adding the NIHSS substantially improved discrimination of the risk of 30‐day mortality in ischemic stroke compared with a model using administrative claims data alone, with an improvement in the c statistic from 0.77 to 0.84.^30^ In this paper, we showed that the addition of the NIHSS improved model discrimination in ICH and SAH, as well as in ischemic stroke (Table 5).

The prediction model that included NIHSS performed well in ICH and SAH despite the absence of several variables that are known predictors of poor outcomes in these populations. In ICH studies hematoma volume, presence of intraventricular hemorrhage, and Glasgow Coma Scale have been found to be independent predictors of poor outcome.^8–16^ In SAH studies clinical grading systems such as the Hunt and Hess scale or the World Federation of Neurosurgical Societies scale,^17–18^ location of the aneurysm,^31^ and extent of subarachnoid blood^17–18^ have been independently associated with poor outcome. Had information on these factors been available in GWTG‐Stroke, it is possible that our ability to predict death in these stroke types would have been further improved. However, our results suggest that risk of death in these stroke types can be predicted well even in the absence of information on these factors. A reason for the excellent discrimination may be that the NIHSS is a good marker of stroke severity in hemorrhagic stroke types as well as in ischemic stroke. Previous studies showed that the NIHSS is a strong independent predictor of outcome following ICH^9,12^ and also following SAH, particularly in SAH patients without depressed consciousness.^32^ Therefore, routine collection of the NIHSS for all stroke types may facilitate risk stratification and allow for hospital‐level stroke risk models with enhanced discrimination.

In our model, we did not adjust for orders for limitation of care or use of palliative care, which would have further increased the discrimination of death.^33^ By design, we chose to adjust only for patient characteristics present on hospital admission to allow our model to be used at the time of admission for the purpose of predicting hospital outcomes and to facilitate better quality of care by allowing hospitals to identify patients with actual outcomes that were better or worse than predicted. Therefore, we chose not to adjust for postadmission events including the quality of care provided or the use of palliative care. This approach follows recommendations from the American Heart Association for risk adjustment suitable for reporting of health outcomes.^34^

To our knowledge, the only other clinical risk score for stroke outcome that has been validated in both IS and ICH is the Six Simple Variable model.^1^ This model was derived in only 530 subjects using data mostly from the subacute period following stroke (median assessment, 4 days after onset; 45% were not admitted to hospital) and did not include data from 50 patients who died before they could be assessed. The predictors modeled were age, living alone, independence in activities of daily living before stroke, verbal component of the Glasgow coma scale, arm strength, and ability to walk. The model was validated in 1330 subjects and showed good discrimination of 30‐day mortality (c statistic, 0.88). However, the number of ICH patients in the validation cohort was small (97/1330, 7.3%). A subsequent independent validation study carried out in hyperacute stroke (<6 hours' duration) showed worse discrimination of 30‐day death (c statistic, 0.73), probably because the model was derived in subacute stroke survivors excluding those who died early. Thus, the Six Simple Variable model may not be optimal for the purpose of making predictions at the time of hospital admission. By contrast, our model was derived using information present at the time of hospital admission, including a large number of ICH patients, and was derived in a contemporary cohort of consecutive acute stroke patients contributed by hospitals across the United States including larger academic and smaller community hospitals.

There are several limitations to this study. Hospital participation in GWTG‐Stroke is voluntary. Although academic teaching hospitals are overrepresented in GWTG‐Stroke, the demographics and comorbidities of the ischemic stroke patients in GWTG‐Stroke are similar to the overall characteristics of US ischemic stroke pateints.^35^ Study data depend on the accuracy and completeness of medical record documentation; however, a data validation audit suggests good reliability.^36^ NIHSS data were often missing, and we cannot be sure that the relationships between predictors and outcome observed in the subset with NIHSS are the same as in the overall study population. Postdischarge information is not collected in GWTG‐Stroke; therefore, we were not able to test whether the risk score predicts postdischarge mortality or functional disability.

In summary, the validated GWTG‐Stroke risk score predicts the risk of dying in the hospital from either IS, ICH, or SAH and appears to better discriminate the risk of in‐hospital death than can be done using administrative data. The predictions generated by the risk score may prove useful to clinicians for counseling patients and their families on prognosis and for identifying groups of patients at highest risk for poor outcomes who may require more intensive monitoring or therapy. Hospitals may find these predictions useful for comparing their observed mortality with their predicted mortality. Automated calculations of mortality risk are now available to participating hospitals via the Web‐based Patient Management Tool, whereas non‐GWTG‐Stroke hospitals could use bedside decision support aids to implement mortality predictions based on our score. Further research will be needed to study how hospitals and individual physicians use this information to guide care.
